# Psychosis and Rectal Bleeding as the Presenting Symptoms of AIDS in an Adolescent Male

**DOI:** 10.7759/cureus.15272

**Published:** 2021-05-27

**Authors:** Rana Harhay, Nicholas J Alexander, Gretell Gomez, Meghan E Jacobs

**Affiliations:** 1 Pediatrics, University at Buffalo Jacobs School of Medicine & Biomedical Sciences, Buffalo, USA

**Keywords:** health care disparities, racial inequities, hiv testing, health inequities, socioeconomic disparities

## Abstract

Socioeconomic status, racial health disparities, and age are some of the many barriers that may confound and delay the diagnosis of human immunodeficiency virus (HIV). Despite the new and highly effective advancements in the treatment of HIV, in the United States, there is a disparity in the rate of timely diagnosis and treatment among Black/African Americans, men who have sex with men, those who suffer from homelessness, and transgendered youth. While there are existing recommendations in place to guide the testing of adolescents for HIV, the rate of testing is highly variable, which can lead to missed diagnoses. We present the case of a previously healthy 17-year-old African American male with psychosis who was initially diagnosed with a primary psychiatric disorder. Three weeks later, the patient presented with rectal bleeding. Further evaluation revealed that the patient was positive for HIV. The psychiatric symptoms were attributed to HIV dementia and rectal bleeding to HIV colitis. To our knowledge, this is the first case report of a patient with HIV dementia and rectal bleeding as the initial presenting symptoms of AIDS. This case demonstrated the potential consequences of variable testing practices for HIV and the devastating sequela that can follow the lack of timely diagnosis.

## Introduction

More than one million people in the United States are currently living with human immunodeficiency virus (HIV) [1]. In 2017, individuals between 15 and 24 years of age comprised approximately 21% of new HIV diagnoses [2]. Despite making up nearly a quarter of new diagnosis, according to preliminary surveillance by the Centers for Disease Control and Prevention (CDC), in 2018, only 9% of students in high school had ever been tested for HIV. The CDC has cited several prevention challenges including inadequate sex education and finding that between 2000 and 2016 the percent of schools in which students were required to receive sex education on HIV prevention decreased by 12% [3]. There are also stark racial disparities that exist in the diagnosis and treatment of HIV. Of all racial and ethnic groups in the United States, Black/African American people account for the highest proportion of new diagnoses of HIV. While only comprising 13% of the population of the United States, Black/African American people made up 42% of new HIV diagnoses in the United States in 2017 [4].

It has also been observed that there is a disparity in the rate of HIV infection among men who have sex with men, those who suffer from homelessness, and transgender youth. The largest proportion of new HIV infections occur among African American young men who have sex with men (YMSM) [5]. Over the last several years, advancement in antiretroviral therapy has helped in both suppressing HIV viral replication and decreasing HIV transmission [6,7]. However, in the United States, adolescents, young adults, and particularly black YMSM are not benefiting from these advancements [8].

## Case presentation

We present the case of a 17-year-old African American male with congenital methemoglobinemia and a family history of schizophrenia who came to the hospital for altered mental status and a 30-pound unintentional weight loss. Upon initial hospital presentation, the patient was altered, confused, and difficult to redirect. His only complaint was throat pain. He denied vomiting, diarrhea, urinary or fecal incontinence, or general recent illness. He denied any sexual activity or drug use.

Upon presentation, he was found to have a low-grade fever but was otherwise hemodynamically stable. Initial blood work-up demonstrated mild leukopenia, no bandemia, normocytic anemia, and transaminitis with aspartate aminotransferase (AST) of 115 unit/liter and alanine aminotransferase (ALT) of 76 unit/liter. Mental status examination revealed a distracted, emotionally labile, tearful young male, and the remainder of the physical examination was normal. Family history was significant for a mother with schizophrenia. The patient was admitted for further work-up of new-onset psychosis.

During this initial hospital stay, the patient underwent a comprehensive interdisciplinary work-up. Given the elevation in liver enzymes, Epstein-Barr virus (EBV) and cytomegalovirus (CMV) antibodies were obtained, which demonstrated a previous EBV infection and an acute CMV infection. The patient underwent an EEG and an MRI of the brain, which were normal. Psychiatry service evaluated the patient and attributed symptoms to new-onset schizophreniform disorder, recommending transfer to an inpatient psychiatric facility.

Three weeks after initial presentation, the patient was urgently transferred from the inpatient psychiatric facility back to the emergency department due to sudden onset of rectal bleeding. This was the first occurrence of rectal bleeding in the patient’s lifetime. History revealed that while at the psychiatric facility, the patient had been started on multiple medications including quetiapine, olanzapine, risperidone, and benztropine, without improvement in psychotic features. During this second emergency department presentation, the patient was ill-appearing and febrile. Laboratory work-up revealed a normal white blood cell count, worsened normocytic anemia, persistent but improved transaminitis with an AST of 74 unit/liter and an ALT of 47 unit/liter, and elevated inflammatory markers. Contrast tomography (CT) of the abdomen and pelvis with contrast was performed, which revealed wall thickening within the sigmoid colon.

Physical examination revealed a catatonic, minimally responsive patient. He endorsed that he felt weak and had significant bradykinesia with parkinsonian-like features. Rectal examination did not reveal foreign objects or masses. The abdomen was mildly tender to palpation. No rashes or lesions were noted. Cardiovascular and respiratory examinations were within normal limits. Given the rectal bleeding, the gastroenterology team was consulted, which recommended an emergent colonoscopy and esophagogastroduodenoscopy (EGD) due to persistent acute rectal blood loss. EGD revealed diffuse esophageal candidiasis. Colonoscopy showed deep ulcerated lesions and thickened mucosa within the rectum. Following review of the chart and with new evidence of esophageal candidiasis on EGD during this second admission, HIV-1 and HIV-2 antigen and antibody screens were sent, and HIV-1 resulted positive. Further work-up revealed that the patient was negative for hepatitis B virus surface antigen and hepatitis C. CD4 count was found to be 123 cells/mm^3^, meeting the criteria for AIDS.

## Discussion

This case highlights the serious outcomes that result from the barriers to the diagnosis and treatment of HIV in relation to age, socioeconomic status, and racial health disparities. Black/African Americans have been disproportionately affected by the HIV/AIDS epidemic [9], and recent statistics show that this disparity has only increased over time [10]. It is well known that prompt medical care during the early stages of HIV can substantially alter a patient’s clinical course [11]. In addition, while individuals of lower socioeconomic status are more likely to report prior testing for HIV [12], they are less likely to receive an early diagnosis when compared to individuals in a higher income status. Furthermore, studies suggest that uninsured HIV-infected patients with Medicaid receive lower quality care [12]. The individual in the case was an adolescent African-American male on Medicaid insurance.

Additionally, this case calls into question the current recommendations for testing children for HIV. The CDC recommends that everyone between 13 and 64 years of age get tested for HIV at least once. For patients at higher risk, the CDC recommends getting tested once a year. [13]. The American Academy of Pediatrics (AAP) recommends that routine screening be offered at least one time to all adolescents between 16 and 18 years in healthcare communities where the prevalence of HIV in the population is greater than 0.1%. In areas where the community prevalence is not as high, the AAP recommends testing for all adolescents who are sexually active or those with any risk factors for HIV [14]. As they are not necessarily mandates, these recommendations leave the discretion of testing up to the physician, which leads to a large degree of variability in testing. For instance, one study analyzing a large cohort of pediatric patients in a primary care network found that 80% of adolescent sexual histories were undocumented, 2.6% of patients were tested for gonorrhea and chlamydia, and only 1.6% were tested for HIV at least once since they turned the age of 13 years [15].

## Conclusions

Every child should have prompt access to the widely available and effective treatments for HIV, but early detection is key for proper treatment. Removing the variability in testing of HIV in the outpatient setting may be achieved by stricter adherence to set guidelines by the AAP and ensuring that children attend well-child checks as scheduled. Furthermore, improving sexual education in public school systems in the United States may also make adolescents more aware of preventative safe sexual practices and the importance of getting tested for sexually transmitted illnesses. Additionally, making parents aware of these guidelines early in adolescence may help improve advocacy for testing. Lastly, all efforts should be made to take a detailed sexual history at every adolescent well-child check. Preventative education and proactive testing can promote safe sexual practices among adolescents and help bridge the socioeconomic, racial, sexual orientation, and age gap in the prevention, diagnosis, and treatment of a disease with devastating sequela.
